# Case studies on the impact of ex-post legislative evaluations in Dutch healthcare: a within and cross-case analysis

**DOI:** 10.1080/13572334.2024.2411480

**Published:** 2024-10-17

**Authors:** Linda J. Knap, Johan Legemaate, Roland D. Friele

**Affiliations:** a Netherlands Institute for Health Services Research (Nivel), Utrecht, the Netherlands; b Tranzo Scientific Center for Care and Wellbeing, Tilburg University, Tilburg, the Netherlands; c Law Centre for Health & Life, University of Amsterdam, Amsterdam, the Netherlands

**Keywords:** Ex-post legislative evaluation, impact, case studies, realist evaluation, mixed methods, cross-case analysis

## Abstract

Globally, ex-post legislative evaluations are becoming increasingly important for understanding how laws function in practice and identifying their limitations and their effects upon stakeholders. This study delves into the impact of ex-post legislative evaluations within the Dutch healthcare system. Building upon insights from previous literature, we aim to refine existing ideas within the field through empirical data. Utilising the realist evaluation method, we examine three distinct case studies followed by a cross-case analysis. Our research underscores that the impact of these evaluations extends beyond policy and politics to the broader societal arena. Our findings also point towards opportunities for strengthening the impact of ex-post legislative evaluations within this broader societal arena. Specifically, we identify strategic phases within the evaluation process through which we aim to maximise impact. Finally, the study emphasises the importance of context awareness, the strategic utilisation of research quality as well as interactional factors for enhancing impact.

## Introduction

Legislation plays a pivotal role in shaping healthcare policies and practices, which, in turn, affect the lives of both providers and patients. The practical effects of legislation often remain unclear until ex-post legislative evaluations (hereafter: legislative evaluations) have been conducted. These evaluations, also referred to in the literature as post-legislative scrutiny, are vitally important for assessing the effectiveness, efficiency, and impact of legislation once it has been implemented (van Schagen, 2020). They also provide opportunities for lawmakers to gather feedback, identify unintended consequences, and make informed adjustments to improve existing laws and policies (Anglmayer & Scherrer, 2020; Caygill, 2019; Kuchava, 2019; van Voorst & Mastenbroek, 2017).

Moreover, legislative evaluations strengthen transparency and accountability within governance, alongside ensuring that decisions are both based upon facts and responsive to the needs of the public (Griglio & Lupo, 2020; Martín, 2016; van Voorst & Zwaan, 2019; Zwaan et al., 2016). In this respect, they can play a crucial role in terms of enhancing the quality of legislation and fostering trust in democratic institutions.

The importance of legislative evaluations is increasingly recognised (European Court of Auditors, 2018). Various countries have entities responsible for these evaluations. For instance, in the United States, the Government Accountability Office evaluates federal programmes, with congressional committees providing oversight. Similarly, in the UK post-legislative scrutiny is conducted by select committees in both the House of Commons and House of Lords (Caygill, 2019; Norton, 2019). In Germany, Bundestag committees oversee legislative implementation and assess their societal impacts (Siefken, 2021). Australia, Canada, and Sweden also have parliamentary committees dedicated to post-legislative scrutiny, testifying to the increased global acknowledgment of the significance of legislative evaluation (Moulds, 2020).

Also approaches to legislative evaluations can vary worldwide. De Vrieze categorises them into passive, informal, formal, and independent scrutinisers. The UK and Switzerland are identified as independent scrutinisers, while the federal parliament of Germany is a passive scrutiniser (De Vrieze, 2020; De Vrieze, 2023). In some jurisdictions, such as Australia, the process is ad hoc, lacking a systematic framework (Moulds, 2020). Conversely, the House of Commons’ systematic approach to post-legislative scrutiny has not yet become a regular part of committee work, and the extent of scrutiny in the House of Lords is also limited (Caygill, 2020). Despite these variations, the need for a structured and deliberate approach to post-legislative scrutiny is clear, as it can lead to meaningful outcomes for citizens (Moulds, 2020).

However, although the importance of legislative evaluations is widely recognised, a research gap remains concerning the actual impact of legislative evaluations and the strategies to optimise this impact. International practices, such as those observed in the European Parliament and parliaments of countries like the UK, Malaysia, and Australia, highlight the importance of post-legislative scrutiny in adding value and enhancing legislative processes. For example, Norton (2019) discusses how the UK Parliament has integrated post-legislative scrutiny to improve legislative outcomes. Additionally, OECD reports (2020, 2021) emphasise the need for systematic reviews of regulatory frameworks to ensure their effectiveness and relevance over time. This broader international perspective underscores the necessity to empirically substantiate the factors influencing legislative evaluation impact and to explore strategies for optimising this impact, thereby learning from diverse legislative environments to refine and enhance the impact of ex-post legislative evaluations globally.

Recognising the importance of a systematic approach to legislative evaluations, the Netherlands has been running the ZonMw Regulatory Evaluation Programme (hereafter: the ZonMw programme) since 1997 to evaluate healthcare legislation and regulations. ZonMw, the Netherlands Organisation for Health Research and Development, is a governmental organisation that funds health research and promotes the use of developed knowledge to improve health and healthcare in the Netherlands. The primary objective of the programme is to enhance the quality of healthcare legislation. To achieve this aim, independent, multidisciplinary research groups are selected to carry out each evaluation, utilising a blend of empirical and legal research methods. Evaluations within the programme can be based on individual regulations or overarching themes that span multiple laws. Oversight of the programme’s implementation ultimately rests with the Committee for Evaluation of Regulations (CER). This long-term programme has resulted in numerous legislative evaluations, the development of a well-defined approach, and the strengthening of the capacity to carry out these evaluations.

To address the research gap on the impact of legislative evaluations initiated by the ZonMw programme, we launched a project investigating the impact of ex-post legislative evaluations in healthcare in the Netherlands. We began with a literature review that highlighted impacts across both policy and political domains, as well as societal domains, with a notable emphasis on policy and political impacts (Knap, van Gameren, et al., 2023). We systematically identified key factors influencing these impacts, emphasising the importance of the political environment and the institutionalisation of evaluation processes, which are beyond researchers’ control (Knap, Friele, et al., 2023). The review also revealed several modifiable factors affecting evaluation impact, such as research quality and researcher-stakeholder interactions. However, the literature primarily consists of expert opinions and case descriptions, lacking empirical data. Many authors discuss the effects of evaluations without empirical support, with expert opinions dominating the discourse (Knap, van Gameren, et al., 2023). Thus, there is a pressing need for empirical research to validate the assumptions from our earlier review (van Aeken, 2011).

Drawing upon both recent research insights and the rich heritage of the ZonMw programme, we aim to answer this need, by delving deeper into three ex-post legislative evaluations in the Netherlands. We answer the following two research questions:

(1) What impact do these evaluations have?
(2) Which factors influence the impact (context, research quality, stakeholder interaction or other factors)?

## Research method

This study adopted a mixed methods approach, which was grounded in realist evaluation. The study focuses on three ex-post evaluations of legislation within the ZonMw programme. The study spanned from March to October 2023. The three cases were selected based on the following considerations: timing (all were at least two years old to allow for impact to unfold), the subject matter (diversity in the ethical or non-ethical nature of the topic), and variation in the frequency of prior evaluations of the law. Guided by these criteria, the study encompassed the following distinctive cases:

- The first evaluation of the Youth Act (published in January 2018)
- The first evaluation of Healthcare, Quality, Complaints and Disputes Act (Wkkgz) (published in February 2021)
- The third evaluation of the Embryo Act (published in March 2021)

It is noteworthy that the researchers in this study formed part of the research group for the Wkkgz and partly for the Youth Act. We consider this to be an advantage in terms of obtaining in-depth information from the researchers’ perspective.

This study comprehensively explores the process from proposal creation during the initiation phase to the dissemination of results in the implementation phase.

This research project underwent a rigourous ethical evaluation by the Ethics Review Board of Tilburg University (ref. no. TSB_RP998), which found no ethical or legal objections to the research.

A comprehensive study protocol detailing the methodology of this study was previously published (Knap, Legemaate, et al., 2023). This methods section provides an overview along with modifications or enhancements to the initial protocol. The study findings were presented in accordance with RAMESES II reporting guidelines for realist evaluations (Wong et al., 2016). A realist evaluation design is well-suited to assessing what works in specific circumstances and for whom. Aligned with this design, the study began with the initial programme theory (IPT) development, which outlines how mechanisms (M) operate in a specific context (C) to achieve certain outcomes (O) (Pawson & Tilley, 1997). The literature review identified research quality (composition and independence of research group, methods used, quality and content of evaluation report) and stakeholder interaction (interaction between researchers and stakeholders, presentation and availability of research results, timing of evaluation) as key and modifiable factors influencing the impact of ex-post legislative evaluations (Knap, Friele, et al., 2023). In the context of realist evaluation methods, these factors represent the mechanisms (M), while the impact(s) of ex-post legislative evaluations are the outcomes (O), all occurring within the context (C) of the legislative evaluation process, such as the characteristics of the law and legislative process, and the political and societal influence. The IPT connects C, M and O and is tested and refined based on the research findings of this study. Together, the IPT and the so called CMO configuration (context, mechanisms and outcomes) provide a framework for unravelling the causal web of conditions underlying the outcomes (Pawson & Tilley, 1997). Table 1 provides an overview of the IPT, and CMO configurations that were used as a framework to study the three ex-post legislative evaluations.

To examine this CMO configuration, we employed a mixed methods approach, consisting of a document analysis, focus group discussions and questionnaires. Given our desire to involve the field in this research, we chose to conduct focus groups and questionnaires. Running concurrently, the three methods addressed identical research questions:

(1) What impact do these evaluations have?
(2) Which factors influence the impact (context, research quality, stakeholder interaction or other factors)?

In the paragraphs below, we provide a detailed description of each data source and the methods used.

### Document analysis

As part of our comprehensive document analysis to address the research inquiries, numerous documents spanning various points of the evaluation process, from initiation to implementation phases of the ex-post legislative evaluations, were scrutinised. Our analysis focused exclusively on visible, objectively, and explicitly stated impact-related information concerning the legislative evaluations.

In our document analysis, several key aspects were emphasised. The impact was assessed through the responses from the Minister of Health, Welfare and Sport (hereafter: the Minister), political figures, and relevant stakeholders in the field. Contextual factors were also considered, such as media attention garnered through the Internet and newspapers, as well as political attention manifested in House of Representatives debates. Stakeholder interaction was evaluated by examining the evaluation report to understand what interactions were sought by the researchers. The quality of the research was scrutinised through the evaluation report, which included the composition of the research group, the recommendations and intended addressees, and the methodologies used, whether qualitative, quantitative, legal, empirical, or a combination thereof. Additional documents from ZonMw, including the programme text and advisory committee opinions, were reviewed to assess awareness of context and impact. The reaction of the Minister was also noted, particularly opinions on the evaluation and subsequent steps in law, policy, or society.

### Questionnaire

A computerised questionnaire was distributed to all stakeholders engaged in the evaluation process, with a distinction being drawn between creators (researchers) and users (policy members, politicians, legal experts, ZonMw advisory committees and a range of relevant societal stakeholders). Consequently, two distinct versions of the questionnaire were devised (see Appendices 1 and 2) but both contained the same topics, all related to impact on one hand and to context, stakeholder interaction, and research quality on the other hand. Respondents could also provide additional topics or factors. Despite substantial points of commonality, the questionnaires exhibited subtle variations as a result of being tailored to the two respective groups. Notably, researchers were asked whether pre-considerations related to the anticipated impact of their ongoing evaluation had been undertaken (see Appendix 2).

The response time for the survey was six weeks, with those who did not complete the questionnaire being reminded on two occasions. The questionnaire was sent to all the researchers in the three cases (*N* = 23) and to 467 users in total. The questionnaire was primarily completed by researchers and relevant societal stakeholders such as healthcare professionals and members of a professional organisation. The cumulative response rate was 45.5%. The questionnaire data were reported using radar charts, merging outcomes from each legal evaluation into one radar chart for each question. This method quantified responses into percentages, which provided a comprehensive understanding of the participants’ feedback distribution. Radar charts were chosen because of their ability to simultaneously present the data from the three evaluations.

### Focus group discussions

A series of five distinct focus group discussions, each lasting one-and-a-half hours, were conducted. These sessions involved an average of six key stakeholders who were directly relevant to the three ex-post legislative evaluations. The participants comprised both creators and users. While joint sessions were organised whenever possible, when scheduling conflicts arose, separate meetings for researchers or users were arranged. In instances involving user-focused conversations, meticulous attention was paid to ensuring that the diversity of the participants was well-balanced, in order to sufficiently represent patients’ interests. The questions that were central to the focus group discussions were aligned with the research questions: (1) What impact do these evaluations have? and (2) Which factors influence the impact?

During the focus group discussions, respondents from the field struggled to think solely about the impact of the legislative evaluation and, in fact, on several occasions responded by referring to the broader context of the impact of the legislation itself. This issue was clarified by the researchers on multiple occasions during the discussions.

### Analysis

The data collected from the three research methods were analysed concurrently. Focusing on actors, we report on both creators (the evaluation researchers) and users, categorised into three groups: policy, politics, and society more broadly.

Initially, a within-case analysis was conducted to identify the unique characteristics of each case study. In line with the research questions of this study, the first author systematically extracted all types of impact and factors influencing impact for each case. This systematic overview produced objective findings from all three research methods. The third author independently conducted this analysis, followed by discussions between the first and third authors to reach a consensus on any points of contention. These findings were then extensively deliberated by all the authors. A detailed overview was created for each case, outlining all the different types of impact and identifying the various influencing factors (see Appendix 3).

Subsequently, the first and third authors interpreted the results for each case study in order to establish connections between the impact and influencing factors. These interpretations were discussed with four external experts during three individual expert meetings. The within-case analysis followed a similar format for each case study. A cross-case analytical approach was then employed to identify similarities and differences between the cases with respect to these topics. At this stage, the emphasis shifted from the respondents’ opinions to the researchers’ interpretation of the within-case data. Based on the cross-case analysis, the CMO configuration was refined to clearly incorporate and highlight the newly acquired insights.

## Results

In this results section, we start by outlining the institutionalisation of legislative evaluations in Dutch healthcare, providing the overarching context within which the three evaluations took place. This is followed by a within-case analysis that describes the specifics of each case separately. Finally, we present a cross-case analysis that examines the three cases together, highlighting comparative insights and overarching themes.

### The institutionalisation of ex-post legislative evaluations

The evaluations we examined were conducted within the ZonMw Regulatory Evaluation Programme, a well-established framework dedicated to assessing healthcare legislation and regulations, overseen by the ZonMw Committee for Evaluation of Regulations (CER). Each evaluation was initiated at the request of the Minister of Health, Welfare and Sport, in accordance with an evaluation clause within the law itself. The CER invited independent research groups to submit a research proposal. In two of the three cases, more than one proposal was received, and these were reviewed and assessed by independent reviewers. Based on these reviews and the answers provided by the research groups, the committee then selected the best research proposal. For each of the subsequent evaluations, an advisory committee was formed on behalf of ZonMw, consisting of members from the CER and experts from the field, in order to oversee the evaluation process. Upon completion, the evaluation report of each evaluation was submitted to the Minister, who provided a written response detailing whether the recommendations proposed were adopted or rejected and specifying the subsequent course of action.

### Within-case analysis

In Table 2, we present the summarised data for all three case studies side-by-side. See Appendix 3 for a more detailed elaboration. We found no factors influencing impact other than those covered by context, research quality, and stakeholder interaction, despite explicitly seeking other factors. However, we did identify additional phases within the evaluation process, outside of the formal evaluation itself. Therefore, we added these additional phases to Table 2. We then consider, for each case, the relationship between impact and the factors influencing impact.

#### Within-case reflection of case 1: evaluation of the Youth Act (2018)

The Youth Act introduced significant changes in a turbulent domain, especially regarding decentralisation, shifting responsibility from the national government to local authorities such as municipalities. There was significant concern in the field about budget cuts and municipal procurement practices. Numerous other studies emerged both during and after the evaluation, potentially limiting its impact as it was overshadowed by this additional research.

The early review, requested by the House of Representatives, restricted researchers from drawing firm conclusions about the law’s effectiveness. However, this early evaluation met the Minister’s need to take control and direct the reform agenda based on the results. During the evaluation, a new cabinet with a new Minister sought greater direction. The evaluation report allowed the Minister to formulate and share their policy vision with the field.

Field stakeholders expressed concerns that researchers’ focus on implementation and opportunities for improvement led to the omission of important topics from the evaluation. This raised questions among societal stakeholders about why certain topics, they considered vital to the law, were not addressed. Furthermore, it raised doubts about whether the Minister genuinely sought a fair evaluation and was open to the practical realities, potentially diminishing its impact. The absence of specifically addressed recommendations and concrete action points may also have contributed towards a reduced impact insofar as no one could be held accountable for the implementation.

Engaging stakeholders prior to, during, and after the evaluation resulted in them having knowledge about the conducted evaluation and its resulting outcomes.

The presentation of the research results during regional and national meetings following the evaluation may have contributed to a greater impact within the field.

#### Within-case reflection of case 2: evaluation of the Wkkgz (2021)

The Wkkgz is a law that is organisational in nature and has a broad scope, and it did not cause unrest within the field. As the law had been in force for five years already, it could be evaluated according to the timing stipulated in the law itself. This time span enabled researchers to draw well-informed conclusions and provide recommendations on the legislation itself. Indeed, several of these recommendations were taken up by the Minister.

The researchers chose to structure the study around five selected healthcare sectors to provide a representative overview of the wide range of areas covered by the Wkkgz. It appears that the impact within these five sectors is greater than in sectors not included in this selection.

Involving respondents from the field and policy circles in an expert meeting at the beginning of the evaluation led to the identification of currently relevant topics and issues, thus aligning the evaluation well with stakeholders’ experiences. By continuing to involve respondents throughout the evaluation process, the impact was thus created within the field. Participation in the evaluation research itself was even considered to be somewhat of an intervention, resulting in new useful insights within the selected healthcare sectors, according to the participants.

The recommendations in the evaluation were primarily addressed to the policy domain and the legislator, which in itself may have contributed to the fact that most of the impact occurred there. Furthermore, researchers opted to formulate practical and concrete recommendations, which were found to be useful within these domains. Finally, the decision of the researchers to present the results during a webinar also likely contributed towards the generation of impact within the field.

#### Within-case reflection of case 3: evaluation of the Embryo Act (2021)

The Embryo Act focuses on a very specific domain where stakeholders know each other, and the lines of communication are short. This facilitated collaboration between the field and national policy, potentially speeding up the uptake of evaluation results. The researchers were deeply embedded in the domain and were considered the appropriate authorities to conduct the evaluation, with some having prior involvement in evaluations of the Embryo Act.

The postponement of the evaluation by the House of Representatives resulted in an evaluation that did not coincide with the implementation actions outlined in the previous evaluation. This may have provided greater room for impact within both the policy and political domains.

Given that this was the third evaluation, the assignment was more specific and refined in scope, building upon previous insights into how the law functioned in practice.

Due to the politically sensitive nature of this law, the researchers were well aware that the political context was crucial for the potential influence of evaluation results on legislation and regulation.

In terms of the design of the evaluation and both the formulation and addressing of the recommendations, the researchers took into account the potential areas in which the evaluation could generate an impact. This, in turn, allowed for maximum impact within relevant areas to be achieved.

The decision of the CER to exclude the societal perspective from the assignment influenced both the focus and scope of the research.

### Cross-case analysis

In this cross-case analysis, we examine the three aforementioned legislative evaluations together, focusing on overarching themes related to context, research quality, and stakeholder interaction in relation to their impact. We aim to uncover discernible patterns across the different cases.

#### Cross-case reflection on impact

Each evaluation generated significant impacts across various domains, as evidenced by the document analysis, focus groups, and the responses to the questionnaire (see Appendix 3 and Appendix 4, question A). For instance, the evaluation of the Youth Act yielded substantial effects on politics, policy, and society more broadly. It prompted immediate parliamentary inquiries and debates, shaped subsequent political discussions, and informed policy reforms. At the societal level, the evaluation was widely disseminated and elicited responses from professional associations focused on improving access to youth care. Unlike the evaluations of the Wkkgz and the Embryo Act, this evaluation did not influence legislation. Although the evaluation of the Wkkgz and Embryo Act led to legislative changes, as shown by the document analysis, this was not always known to members of the professional field and was therefore not always mentioned in the questionnaire.

The evaluation of the Wkkgz also influenced policy, resulting in legislative revisions and recommendations implemented at both the ministry and regulatory levels. It also generated broader societal impacts, with professional associations raising concerns and dispute resolution bodies increasing their level of cooperation with one another. Additionally, the Inspectorate commissioned further research prompted by the evaluation findings.

Similarly, the evaluation of the Embryo Act had a significant impact on policy, legislation, and political circles. Specifically, it led to amendments, consultations, and recommendations addressed within ministries, thus indicating its influence on legislative processes and political agendas. However, according to the questionnaire, the greatest impact was observed in the societal domain amongst healthcare providers (see Appendix 4, question A). The reason for this is presumably because it is a tightly defined domain in which professionals in the field maintain close contact with policymakers at the ministry.

Below, we continue the cross-case analysis for each factor. First, we describe the relevant contextual factors, followed by research quality and stakeholder interaction. We will then illustrate the connections between these factors and the observed impact in the discussion section.

#### Cross-case reflection upon the influence of context on impact

The first contextual factor shared by all three evaluations is that the legislative evaluation process is structured within the ZonMw programme, which remains consistent across all cases. An institutionalised system like the ZonMw programme not only assures overall evaluation quality but also facilitates impact in key domains, such as policy and politics, including legislative revisions, agenda-setting, and requests for further research. This can be seen as a ‘main route’, reflected in the predominant focus of existing literature.

Secondly, the timing of the evaluations, determined by the Minister, establishes a more concrete context for each evaluation. In all three cases, the initiative for the legislative evaluations came from a clause in the law. However, in two of the evaluations, the House of Representatives intervened to adjust the timing, advancing and postponing the evaluations of the Youth Act and Embryo Act, respectively. This timing significantly influenced the dynamics of each legislative evaluation, shaping the focus and mandate at hand. For instance, in response to the adjusted timing, the evaluation of the Youth Act concentrated on observing the evolving process of change in response to this act. By advancing the evaluation, there was ongoing momentum and an evolving situation. Consequently, no firm statements could be made by the evaluation researchers about either the operation or texts of the law; instead, they could only discuss the ongoing process of change. Additionally, no changes were made to the legal text based on this evaluation. On the other hand, this allowed the Minister to respond effectively to the findings of the evaluation and adjust their policy accordingly. However, the societal field questioned the relevance of the evaluation to their daily practice.

Conversely, delaying the evaluation of the Embryo Act potentially increased its impact on the policy and political spheres, insofar as it provided ample time for the implementation of prior recommendations from the previous Embryo Act evaluation and garnered attention for the latest evaluation results and recommendations. Being the third evaluation also solidified the Minister’s mandate. That is to say, from the previous Embryo Act evaluations, it was clear which topics needed further research so this could be a focused evaluation with potentially more impact. A more neutral example was observed in the Wkkgz, where the law was already sufficiently integrated, and the five-year timing clause in the law could be adhered to.

Thirdly, each evaluation took place within a unique context, influenced by various factors such as the type of law, its function, and the political and social climate. For example, the Embryo Act was highly politicised because of ethical and religious issues, whereas the Youth Act was hotly debated in the field due to its impact on the daily activities of youth care providers and municipalities. Indeed, the law had such a substantial impact within the field that the chances of the evaluation itself making a notable impact were reduced. In contrast, the Wkkgz is a broad law that is applicable to a very large group of healthcare providers but has more of an organisational character. Thus, the unique context of the legislation (which is partly shaped by the legislation itself) determines the evaluation’s context and potential impact.

#### Cross-case reflection on the influence of research quality on impact

There were no meaningful differences observed in the quality of the evaluations with respect to both the research group or the research itself. The ZonMw procedure ensures a minimum quality standard for each evaluation. The respondents’ perceptions of the research group’s quality resonate with this assurance, as they assumed that it was maintained through the ZonMw procedure. The evaluation researchers were viewed as the appropriate authorities.

Comments from users across all three cases indicate that the choice of evaluation topics significantly influences its impact. Specifically, selecting relevant topics in the initial phase of the evaluation process can enhance its impact by aligning with the needs and current interests of stakeholders. This alignment can be achieved either by conducting research in a well-defined domain where policy and practice are closely interconnected and experts are actively involved, as seen in the evaluation of the Embryo Act, or by organising an expert meeting at the start of the evaluation, as done with the Wkkgz evaluation. Conversely, a lack of alignment with field interests, as observed in the evaluation of the Youth Act, can lead to missed opportunities for achieving maximum impact. In this case, the evaluation was aligned with the needs of the Minister, resulting in an impact within that specific domain.

In addition to topic selection, the sharpness of the recommendations and their targeted addressing to specific stakeholders were also reported in all three cases as being essential for generating impact in the relevant places.

#### Cross-case reflection on the influence of stakeholder interaction on impact

Across the three case studies, we observed a relationship between the involvement of stakeholders—both prior to, during, and after the evaluation—and the resulting impact. Specifically, various stakeholders were engaged, including the Minister, policy members, healthcare providers, patients, professional associations, municipalities, and the inspectorate. These parties were involved at different stages. The Minister’s role was primarily as the commissioner of the evaluation, participating before and after its execution, while respondents actively engaged during the evaluation process. Across the three cases, we observed that the interaction between researchers and stakeholders began with the engagement between researchers and the CER, acting on behalf of the Minister, to commission the evaluation and reach out to potential research groups to write proposals to conduct the evaluation. Upon receiving the commission, researchers engaged with stakeholders involved in the practical application of the law. The respondents in all three cases reported that the evaluation resulted in new knowledge and insights, while in some instances, as seen in the Wkkgz evaluation, stakeholders began to implement insights even during the evaluation process. The cases thus demonstrate that involvement in the evaluation increases the relevance for stakeholders, thereby enhancing the likelihood of impact being generated.

During the assessment and dissemination phase, researchers had the opportunity to present their findings to a wider audience. In all three cases examined, the evaluations were published on websites, whilst researchers also authored one or more scientific articles. Additional steps were also taken, including engaging in discussions with field parties and organised webinars or conferences by invitation. However, it remains unclear if these efforts resulted in additional impact. What the respondents did deem to be important, though, was making the research results accessible by creating a practical version or summary that was suitable for the broader field.

### Additions to CMO configuration

This research was guided by the framework of an initial programme theory and a CMO (Context-Mechanism-Outcome) configuration (see Table 1). The CMO configuration incorporates mechanisms that influence the impact of legislative evaluations within their contextual parameters. The first mechanism addresses research quality (under A), while the second focuses on stakeholder interaction (under B). Based on the data collected in this study, we have refined and expanded our understanding of the relevance and interrelatedness of context, research quality, stakeholder interaction, and impact. Additional insights, reflecting the main findings of this study, are highlighted in italics in Table 3.

## Discussion

In the discussion, we sequentially reflect upon the two research questions of this study in relation to impact, context, research quality, and stakeholder interaction, as well as the outcomes from the CMO configuration in order to address the research questions posed in this study. We also reflect on strengths and limitations and finish with some concluding remarks.

### Answering research questions

#### Research question 1: what impact did these evaluations have?

We did find evidence that the impact of legislative evaluations within healthcare extended beyond the policy and political domains into the societal domain, which is also a crucial actor when it comes to legislation. Especially in the healthcare sector, citizens, professionals, and healthcare organisations are directly affected by healthcare-related legislation. Moreover, both healthcare providers and patients play an important role in achieving the goals of the law. By explicitly focusing on the potential impact of legislative evaluations within the societal domain, we have thus observed various types of impact. Specially, we discovered that the field takes note of the evaluation, spreads the evaluation on websites or in newsletters amongst its members, and in some instances also acts upon the evaluation. For example, in response to the Wkkgz evaluation, dispute resolution bodies sought each other out and held discussions. It was also reported that other (government-affiliated) organisations acted upon the evaluation results, such as the example of municipalities responding to the evaluation of the Youth Act and the inspectorate. Although these were not the primary addressees in the evaluation, in practice, they turned out to be relevant parties with respect to working with the results. Whilst international literature often focuses narrowly on impact within policy and political domains, such as legislative revisions and the strategic use of evaluation findings in politics (Knap, van Gameren, et al., 2023), only a few studies have addressed the broader impact of legislative evaluations in practice. For instance, research has shown that legislative evaluations contribute towards enhancing the public’s understanding of laws (Klein Haarhuis & Niemeijer, 2009) and facilitates greater democratic debate (van Aeken, 2018). Despite the societal domain being identified as a potential user of legislative evaluations (Hendriks, 2000; Poptcheva, 2013), empirical research with respect to its impact remains limited. Our study shows that these evaluations do generate societal impact, in spite of the fact that the societal field was not the primary focus of these three evaluations. Hence, paying greater attention to ex-post legislative evaluations within both academic literature and daily practice can increase the societal impact of legislative evaluations. Failing to recognise the relevance of the societal domain as a potential impact area, risks overlooking opportunities for potential impact. The impacts generated in this field are incredibly diverse, and we have observed, in particular, that this field has taken note, disseminated the results and, albeit to a lesser extent, field parties have engaged with the evaluation results.

The observations regarding the societal field questioning the relevance of evaluations to their daily practice and the lack of alignment with field interests underscore critical issues in understanding the impact of legislative evaluations. Direct beneficiaries, such as healthcare providers and municipalities, play a crucial role in the practical application of legislative frameworks. For instance, health-related laws serve an instrumental function by providing a structured framework within which complaint handling can occur, enabling the optimisation of processes and ensuring better service delivery. In the context of youth services, decentralisation has granted municipalities significant freedom to design and implement their processes. However, the field expressed a desire for the evaluation of the Youth Act to address other subjects more relevant to their immediate needs, illustrating the two-way interaction between evaluations and practice. Practitioners can leverage evaluations to highlight and advocate for issues they consider important, thereby strengthening their position and ensuring that evaluations are not only retrospective analyses but also proactive tools for driving improvements. This alignment between legislative evaluations and the practical needs of the field can enhance the overall impact, fostering more responsive and effective legislative frameworks that better serve the public.

#### Research question 2: how does the context, research quality and stakeholder interaction influence this impact?

We will answer this question per factor (context, research quality and stakeholder interaction).

#### The influence of context on impact

Context influences impact in manifold ways. This research highlights that context creates a window of opportunity while simultaneously closing others for the potential impact of a legislative evaluation. Our findings add two new contextual insights: institutionalisation and timing (see Table 3). In the cases studied, the context of the evaluations is shaped by several elements, ranging from the institutionalisation of the ZonMw programme to the specific features of the law being evaluated and its surrounding environment. Legislative evaluations have also been institutionalised in other countries. The exact manner of institutionalisation varies but typically involves government agencies, parliamentary committees, and independent bodies analysing laws for their effectiveness and impact. These mechanisms support evidence-based governance, and earlier literature has endorsed the merits of institutionalising this process (van Humbeeck, 2000). Institutionalising ensures that there is a set system that is the same for all evaluations within that system. This institutionalisation, in turn, paves a direct route towards impact, primarily within the policy and political domains with a specific focus on legislative revision. It also supports institutional learning on how to conduct evaluations. While institutionalisation is a consistent factor across all evaluations, we have identified a new contextual factor that varies significantly from evaluation to evaluation and influences their potential impact: the timing of the evaluation. In two of the cases, explicit decisions were made regarding the timing of the evaluation. For instance, in the evaluation of the Youth Act, the decision to conduct the evaluation prematurely reduced the availability of conclusive results regarding the legislation’s effectiveness. This served to limit the potential impact of the evaluation with respect to improving the legislation. Conversely, in the case of the Embryo Act, postponing the evaluation enhanced its impact. Besides timing, the political and social landscape also varied for each of the evaluations examined, which affected their potential impact. In the evaluation of the Youth Act, for example, we saw extensive debates about budget cuts and municipalities’ purchasing strategies that were covered only to a limited extent in the evaluation but played an important role in the daily practice of youth care organisations and professionals (see Appendix 4, question B). In the evaluation of the Embryo Act, there was a highly politicised debate with strong opinions about what could and could not be discussed, which, in turn, defined the options for impact. Despite the fixed nature of these contextual factors and the predefined framework within which evaluation researchers operate, we found that the topics covered in the evaluation matched the scope for actual impact offered by the context. In this case, researchers demonstrated a keen awareness of the politicised landscape within which the evaluation results would be situated, as well as the likelihood of the recommendations being implemented as a result of the evaluation. As a researcher, being aware of the fixed context and anticipating it appropriately can open up windows for greater impact.

#### The influence of research quality on impact

This study examined how the quality of both the research group and the research itself contributed to the impact of the three legislative evaluations. Our study added refined insights across all three subcategories of research quality. We found that alongside diversity in composition and the independence of the research group, the authority of its members also plays an important role. When respected individuals are involved in evaluations, the acceptance of results tends to be expedited.

In all cases, both the evaluations and composition of the research groups were rated as good (see Appendix 3 and Appendix 4, question C). While institutionalisation of legislative evaluations within the ZonMw programme implies inherent quality, our study highlights that researchers can make choices that directly influence both the quality and impact of evaluations. This study emphasises that researchers can select specific methods and tailor the research setup, significantly affecting potential impact. For example, in evaluating the Youth Act, researchers used a mixed-method approach combining quantitative surveys with qualitative interviews of youth service providers, local officials, and community leaders. This inclusive method provided comprehensive insights into the Act’s implementation challenges and successes, enabling targeted recommendations that resonated with local stakeholders and enhanced the study’s impact. All cases indicate that aligning research topics with the interests of stakeholders, and not only those of the Minister, is crucial for improving the relevance of the evaluation for the field. Additionally, this study has demonstrated that it can be valuable to specify and address recommendations to the appropriate parties, which is also within researchers’ control (see Appendix 3 and Appendix 4, question E). Finally, it is important to consider the specific addressee responsible. For example, in the case of the Youth Act, the relevant addressee shifted from the national government to the municipal government. The researchers’ decision not to address the recommendations was not positively evaluated by the respondents in our focus groups (see Appendix 3). Addressing recommendations allows these addressees to be held accountable.

#### The influence of stakeholder interaction on impact

This study has provided examples of interactions between researchers and stakeholders that contribute towards the impact of legislative evaluations. Beyond presenting and making research results available after the evaluation, our research underscores the significant potential of early stakeholder involvement in the evaluation process. Specifically, this study has shown that involving stakeholders early on, by identifying issues that are relevant to them in practice and by engaging individuals as active respondents in the research, can help to generate impact even during the evaluation process. For example, during the Wkkgz evaluation, participants reported that by participating in discussions during the evaluation, they were already working with insights gained during these discussions. We therefore believe that involving people throughout the process increases the likelihood of impact. In addition, it is clear that greater attention needs to be paid to the potential impact within the field and more needs to be done to reach out to the field.

While dissemination after the evaluation appears to be crucial, a less visible impact was observed from these actions. Our research also emphasises the importance of making the evaluation accessible to a broader audience by creating, for example, a practical version or a summary targeted at those within the field. While existing literature primarily focuses on communication after the evaluation, our research indicates that communication before and during the evaluation is equally important for impact.

### Strengths and limitations

This study marks the first systematic effort to assess the impact of ex-post legislative evaluations in the Dutch context, drawing on insights from international literature. Our innovative approach involves all stakeholders engaged in evaluating legislation, shedding light on previously overlooked dynamics. While we acknowledge the significance of societal impact, our study did not directly investigate this field, which presents opportunities for future research. Distinguishing between the impact of legislation and that of legislative evaluation poses challenges, as not all of these aspects are easily observable or quantifiable.

Our direct involvement in two of the evaluations ensured a thorough examination, with findings transparently reported to mitigate any claims of bias. We conducted three case studies, each displaying significant diversity. However, there is a clear need for additional research in different contexts, as all three cases were within the evaluative framework of the ZonMw programme. The realist evaluation method was chosen for its emphasis on contextual nuances, aligning with our goal to comprehensively explore specific contexts, mechanisms, and outcomes. A detailed study protocol was developed to rigourously execute this method. As part of the realist evaluation, we conducted a literature review, which revealed a limited presence of empirical research. Despite this, we developed a conceptual framework based on existing ideas about the impact from this review. While the review underscores the importance of empirical studies like ours, it also points out a limitation: our use of this framework may have led to a perspective that was too narrowly defined in the case studies, despite our efforts to remain open to emerging themes outside the framework.

In this study, our aim was to identify the impact of legislative evaluations and the factors influencing them. To achieve relevant results, we focused on modifiable factors within the case studies. However, this emphasis on modifiable factors, which can influence impact, also represents a limitation of our study as it necessitated a narrowly defined perspective.

The insights gained primarily focus on Dutch healthcare, but they may offer practical information for other countries considering or conducting similar evaluations, though specific examples are not provided in this study. The findings from this study offer important implications for other countries seeking to enhance the impact of their legislative evaluations. Regardless of the responsible entity or the approach they currently employ, different countries can learn from our results. By refining the CMO configuration to include additional contextual factors, research quality indicators, and stakeholder interaction mechanisms, other countries can adopt a more nuanced approach to legislative evaluations. For instance, institutionalising the evaluation process, as observed in the studied ZonMw programme, ensures consistency and supports evidence-based policy-making. Countries can also benefit from considering the timing and political landscape surrounding evaluations to maximise their impact. Furthermore, ensuring the independence and methodological rigour of research teams, alongside targeted and inclusive stakeholder engagement, can significantly enhance the relevance and uptake of evaluation findings. As demonstrated, broadening the focus to include societal impacts beyond political and policy domains can lead to more comprehensive and actionable insights. These practices can help other countries improve their legislative frameworks, fostering greater public understanding, democratic debate, and overall societal benefits.

This wider applicability bolsters the value of our research by informing and improving evaluation processes across diverse contexts.

### Conclusion

This study contributes to the existing literature by shedding light on the global landscape of ex-post legislative evaluations, revealing a significant gap in empirical research with respect to their impact. It emphasises that current structures primarily target influencing the policy and political domains, whilst overlooking the potential impact in the societal domain, which is particularly crucial in fields like healthcare. Our research underscores the importance of involving stakeholders not merely as sources of information but rather as active participants to enhance the relevance and impact of evaluations.

Researchers can proactively make choices throughout the evaluation process in order to align the content with the field, involve stakeholders, and respond to the evaluation context, thus contributing to impactful legislative evaluations. Despite the challenges involved, ignoring this opportunity would represent a missed chance to create a meaningful impact through legislative evaluations. In this respect, our findings provide valuable insights into the complex dynamics of ex-post legislative evaluations, laying a crucial foundation for informed decision-making and potential policy enhancements within healthcare settings.

## Supplementary Material

Supplemental data for this article can be accessed online at https://doi.org/10.1080/13572334.2024.2411480

Supplemental material

## Figures and Tables

**Table 1:** Initial programme theory (Knap, Legemaate, et al., 2023). Initial programme theory Devoting attention to research quality and interaction during the evaluation process affects the impact of an ex-post legislative evaluation

Contexts	+ Mechanisms	= Outcomes
Characteristics of the law and legislative process Evaluation initiation and function and openness to the evaluations results Political and societal influence	A) Research quality: Composition and independence of research group, and Methods used Quality and content of evaluation report B) Stakeholder interaction: Interaction between researchers and stakeholders Presentation and availability of research results Timing of evaluation	Impact of ex-post legislative evaluations within: The legislative community The policy sphere The society more broadly

**Table 2:** Summarised data of the three case studies – within case analysis.

	Case 1 – Evaluation of the Youth Act	Case 2 – Evaluation of Wkkgz	Case 3 – Evaluation of the Embryo Act
**Impact**	Impact within the political sphere: parliamentary questions, extensive political debate and amendment stemming from the evaluation. Impact within policy circles: agenda setting by the Minister. Impact within society: The evaluation was widely shared online, efforts by field parties were made to improve access to youth care and written responses were sent from professional associations to the House of Representatives. No impact upon legislation.	Impact within policy circles: the Minister planned to amend various aspects of the law and recommendations were implemented at the policy level as part of ministry and regulatory oversight. Impact within society: The evaluation was widely shared online, with professional associations expressing concerns in a letter to the House of Representatives. Dispute resolution bodies actively engaged in discussions over the evaluation results. Additionally, the Health and Youth Care Inspectorate commissioned further research into patient involvement after adverse events in Dutch hospitals, partly influenced by the evaluation’s findings.	Impact within policy circles: the Minister announced two amendments to the law. Impact within the political sphere: The House of Representatives conducted an internet consultation, sought advice from the Health Council, and addressed recommendations within ministries. Additionally, a coalition agreement outlined intentions and plans regarding specific evaluation recommendations. Impact within society: The evaluation was widely shared online. The societal impact was evident amongst stakeholders who were directly involved in the domain of the Embryo Act. A research institute produced a report with remarks and criticisms in response to its findings.
**Context**	First evaluation. New law brought about systematic change. Timing brought forward by two years. Unrest in the field regarding the law, many dynamics resulting from the legislation. A lot of other studies emerged at the same time,	First evaluation. Broad and organisational law that resulted in new obligations. Timing in line with evaluation clause. No unrest in the field regarding the law. Evaluation was carried out during COVID-19 pandemic.	Third evaluation. Existing specific law. Timing extended. No unrest within the field regarding the law, very delimited domain.
**Research quality during the execution**	Researchers focused on legal framework and the implementation. Ministers’ transparency in conducting a fair evaluation was doubted due to their exclusion of pertinent field-related topics. The evaluation included 21 recommendations without specifying the recipients	Researchers focused on five exemplary sectors in healthcare, rather than covering all sectors. Recommendations were made practical and concrete, focusing on specific laws and policies, and were deemed to be beneficial by the field. The evaluation included 32 recommendations aimed at seven groups of addressees (especially legislators and policy makers).	Researchers focused on six themes, including unresolved issues from previous evaluations and the future viability of the law. Both researchers and users emphasised the importance of researchers’ authority and independence. Some researchers had prior involvement in Embryo Act evaluations. The evaluation included 12 recommendations, which were directed at specific addressees (especially the legislature).
**Research quality after the execution**	CER praised the report but called for more assertive findings and recommendations. Field respondents rated both the quality of the research group and the quality of the evaluation as good in the questionnaire. Field emphasised the evaluation report’s good summary.	CER praised the report but highlighted some limitations, including a low response rate, too much focus on professionals, and a lack of sharpness in the recommendations. Field respondents rated both the quality of the research group and the quality of the evaluation as neutral to good in the questionnaire.	CER praised the report but called for the adjustment of certain formulations in the recommendations. Field respondents rated the quality of the research group as good to very good and the quality of the evaluation as good in the questionnaire.
**Stakeholder interaction during the execution**	Various stakeholders, including legal perspectives, municipalities, clients, and youth care were engaged. Participants had ample opportunity to give input despite the limitations of the evaluation. Participants kept informed about the results. However, participant involvement in preparation, setup, completion, and meetings was either minimal or entirely lacking.	An expert meeting was organised at the start to identify any practical issues. Relevant stakeholders were involved in online focus groups and interviews during the evaluation as a result of the COVID-19 pandemic. Participants noted that their involvement in the research led to increased interest in the results and the follow-up of the research. It provided them with food for thought, and sometimes led to the initiation of processes within their own organisation. They felt that participating in the evaluation research allowed them to address relevant themes to varying degrees. From the field questionnaire, it was found that stakeholders were involved in the preparation, execution, and informed about the outcomes and meetings, but were either less or barely involved in the setup and completion of the research.	Relevant Dutch and foreign stakeholders were involved during the evaluation, including professional and patient organisations. Prior to the evaluation and formulation of the recommendations, the researchers took into account the chances of implementation. According to the researchers, the way that recommendations are formulated is important for impact. From the field questionnaire, it was found that participants were involved in the preparation, design, and execution, and were informed about the results, but were either less or barely involved in the conclusion and meetings.
**Stakeholder interaction after execution**	Evaluation report was made available online. Researchers presented the results at regional and national meetings. Researchers authored a scientific article. Not all researchers viewed generating impact as their responsibility; after completion, they let go of the research.	Evaluation report was made available online. Researchers presented the results at a webinar. Researchers authored a scientific article. Researchers distributed the report via email to stakeholders. A patient council network requested a simplified report for patients due to both its length and technical nature.	Evaluation report was made available online. Researchers were invited to present their results at a conference. Researchers authored a scientific article. Not all the researchers viewed generating impact as their responsibility; after completion, they let go of the research.

**Abbreviations:**CER: Committee for Evaluation of Regulations; Minister: Minister of Health, Welfare and Sport; Wkkgz: Healthcare, Quality, Complaints and Disputes Act; ZonMw: Netherlands Organisation for Health Research and Development.

**Table 3:** Updated framework (programme theory and CMO configuration). Initial programme theory: Devoting attention to research quality and interaction during the evaluation process affects the impact of an ex-post legislative evaluation. *Updated programme theory:* *Anticipating contextual factors during the evaluation process, along with prioritising research quality and stakeholder interaction, enhances the impact of an ex-post legislative evaluation on policy, politics and society*.

Contexts	+ Mechanisms	= Outcomes
*Institutionalisation of the evaluation process* Characteristics of the law and legislative process Evaluation initiation *(including timing and the number of previous evaluations),* function and openness to the evaluations results Political and societal influence	A) Research quality: Composition, *authority* and independence of research group Methods used *and research setup* Quality and content of evaluation report *(topic selection, formulating and addressing recommendations)* B) Interaction: Interaction between researchers and stakeholders Presentation and availability of research results Timing of *involving stakeholders (prior to, during and after* the evaluation)	Impact of ex-post legislative evaluations within: The legislative community *(within political and policy domains)* *Politics* The policy sphere The society more broadly

Updates, based on the findings of the three evaluations, shown in italic.
