# Supplementary material for: Case studies on the impact of ex-post legislative evaluations in Dutch healthcare: a within and cross-case analysis
Source: J Legis Stud. Author manuscript; Available in PMC 2024 Oct 29. (PMC7616756; doi:10.1080/13572334.2024.2411480)
Supplement: Supplemental material [file EMS199603-supplement-Supplemental_material.zip › Appendix_1.docx]

# **Appendix 1 – Questionnaire for the creators**

**Questionnaire for the creators**

*Translated to English, (x) refers to specific law*

1. The context in which the evaluation of (x) took place.

Would you like to answer the following statements regarding the context of the evaluation of (x)?

1. During the evaluation of (x), there was a highly active **political debate** about one or more aspects of (x).
2. During the evaluation of (x), there was a highly active debate amongst national and municipal **policymakers** about one or more aspects of (x).
3. During the evaluation of (x), there was a highly active debate within **(depending on the law, a relevant field party is mentioned)** about one or more aspects of (x).

Options: No / Somewhat / Yes / I don't know

1. In your opinion, has the evaluation of (x) had an impact, and if so, in what way? *Multiple answers possible*

- The evaluation has provided more knowledge and insight into the points where the Healthcare Insurance Act works well
- The evaluation has provided more knowledge and insight into the points where the Healthcare Insurance Act is not working so well
- The evaluation has led to adjustments in (x)
- The evaluation has influenced government policy on (x)
- The evaluation has led to a broader discussion in the field about the way (x) came about or the principles of (x)
- The evaluation has led to discussion within the political arena
- The evaluation has influenced the policy of healthcare organisations
- The evaluation has influenced healthcare professionals
- Other, namely:
- The evaluation did not have an impact in any way
- Do not know

1. The quality of the evaluation of (x)
2. Can you provide an assessment of the quality of the evaluation of (x)?
3. Can you provide an assessment of the composition of the research group that evaluated (x)?

Options: Very bad / Bad / Neutral / Good / Very good / I don't know

1. The interaction between the researchers and the stakeholders in the evaluation of (x)

Would you like to respond to the following statements regarding the interaction between the researchers and the stakeholders in the evaluation of (x)?

1. Relevant individuals from the field and/or policy sphere were actively involved in the preparation of the evaluation of (x).
2. Relevant individuals from the field and/or policy sphere influenced the design of the evaluation of (x).
3. During the execution of the evaluation of (x), relevant individuals from the field and/or policy sphere were actively involved, such as, for example, as respondents, in focus group discussions, or as experts.
4. Relevant individuals from the field and/or policy sphere actively contributed to the finalisation phase of the evaluation of (x).
5. Relevant individuals from the field and/or policy sphere were actively informed about the outcomes of the evaluation of (x).
6. Relevant individuals from the field and/or policy sphere attended meetings in which the outcomes of the evaluation of (x) were shared.
7. The results and recommendations of the evaluation of (x) were well-aligned with the needs of the field and/or policy sphere.
8. What factors do you think played a role in the impact generated by the evaluation of (x)? *Multiple answers possible*

- The composition of the research group
- The quality of the research
- The fact that the field was actively involved in the study
- The fact that the researchers formulated recommendations
- The relevance of the results and/or recommendations of the legal evaluation for the field
- The political attention paid to the results and/or recommendations of the legal evaluation
- The fact that the researchers actively disseminated the results through, for example, a webinar, gave presentations and wrote (scientific) publications
- The fact that others, such as the media or stakeholders, paid attention to the results and recommendations of the legislative evaluation
- Other, namely:

1. During the design or implementation of the research, was there consideration given to generating impact through the evaluation of (x)?

- Yes
- No
- I don't know

1. What efforts were made by the researchers to generate impact through the evaluation of (x)?
2. What more could have been done by the researchers to increase the impact generated by the evaluation of (x)?
3. Would you like to receive the results of this research? If so, please provide your email address here:
