# Supplementary material for: Case studies on the impact of ex-post legislative evaluations in Dutch healthcare: a within and cross-case analysis
Source: J Legis Stud. Author manuscript; Available in PMC 2024 Oct 29. (PMC7616756; doi:10.1080/13572334.2024.2411480)
Supplement: Supplemental material [file EMS199603-supplement-Supplemental_material.zip › Appendix_2.docx]

# **Appendix 2 - Questionnaire for users**

*Translated to English, (x) refers to specific law*

Impact of the evaluation of (x).

Legislative evaluations can have an impact in various ways. First, people take note of the results and recommendations. Another form of impact can be that people form their own opinions about them and potentially share them with others. Alongside this, people may also engage with the results and/or recommendations of a law review in their work. We would like to hear from you about what you have done with the results and/or recommendations of the evaluation of (x).

1. Are you familiar with the evaluation of (x)? Multiple answers are possible

- Yes, I have begun working with the results/recommendations of the evaluation in the following way:
- Yes, I have begun working with the results/recommendations of the evaluation in the following way:
- Yes, I have discussed it with others
- Yes, I have read the evaluation
- Yes, I received information about (some of) the outcomes of the evaluation
- Yes, I have heard about it
- No, I am not familiar with the evaluation
- No, I am not familiar with the evaluation

*If you answered no, then move onto question 1.1 / if you answered a variant of yes, then move onto question 2*

- 1. In your opinion, what should have happened so that the evaluation of (x) would have come to your attention?

*Open field*

- 1. At what point should this have happened?
- Prior to the evaluation itself
- During the execution of the evaluation study
- Upon completion of the evaluation study
- Do not know

1. Can you indicate what the main reason was for you not beginning to work on the results/recommendations from the evaluation of (x)?
2. The context in which the evaluation of (x) took place.

Would you like to answer the following statements regarding the context of the evaluation of (x)?

1. During the evaluation of (x), there was a highly active **political debate** about one or more aspects of (x).
2. During the evaluation of (x), there was a highly active debate amongst national and municipal **policymakers** about one or more aspects of (x).
3. During the evaluation of (x), there was a highly active debate within **(depending on the law, a relevant field party is mentioned)** about one or more aspects of (x).
4. In your opinion, has the evaluation of (x) generated an impact, and if so, in what way? *Multiple answers are possible*

- The evaluation has provided more knowledge and insight into the points where the Healthcare Insurance Act works well
- The evaluation has provided more knowledge and insight into the points where the Healthcare Insurance Act is not working so well
- The evaluation has led to adjustments in (x)
- The evaluation has influenced governmental policy on (x)
- The evaluation has led to a broader discussion in the field about the way (x) came about or the principles of (x)
- The evaluation has led to a discussion in the political arena
- The evaluation has influenced the policy of healthcare organisations
- The evaluation has influenced healthcare professionals
- Other, namely:
- The evaluation did not have an impact in any way
- Do not know

1. The quality of the evaluation of (x)
2. Can you provide an assessment of the quality of the evaluation of (x)?
3. Can you provide an assessment of the composition of the research group that evaluated (x)?
4. The interaction between the researchers and the stakeholders in the evaluation of (x)

Would you like to respond to the following statements regarding the interaction between the researchers and the stakeholders in the evaluation of (x)?

1. Relevant individuals from the field and/or policy sphere were actively involved in the preparation of the evaluation of (x).
2. Relevant individuals from the field and/or policy sphere influenced the design of the evaluation of (x).
3. During the execution of the evaluation of (x), relevant individuals from the field and/or policy sphere were actively involved, such as, for example, as respondents, in focus group discussions, or as experts.
4. Relevant individuals from the field and/or policy sphere actively contributed to the finalisation phase of the evaluation of (x).
5. Relevant individuals from the field and/or policy sphere were actively informed about the outcomes of the evaluation of (x).
6. Relevant individuals from the field and/or policy sphere attended meetings in which the outcomes of the evaluation of (x) were shared.
7. The results and recommendations of the evaluation of (x) were well-aligned with the needs of the field and/or policy sphere.
8. What factors do you think played a role in the impact generated by the evaluation of (x)? *Multiple answers possible*

- The composition of the research group
- The quality of the research
- The fact that the field was actively involved in the study
- The fact that the researchers formulated recommendations
- The relevance of the results and/or recommendations of the legal evaluation to the field
- The political attention paid to the results and/or recommendations of the legal evaluation
- The fact that the researchers actively disseminated the results through, for example, a webinar, giving presentations and writing (scientific) publications
- The fact that others, such as the media or stakeholders, paid attention to the results and recommendations of the legislative evaluation
- Other, namely:

8. In what way could the impact of the evaluation of (x) been increased for you?

9. From which position/role did you complete this questionnaire?

10. Would you like to receive the results of this survey?

If so, please provide your e-mail address here:
