# Supplementary material for: Case studies on the impact of ex-post legislative evaluations in Dutch healthcare: a within and cross-case analysis
Source: J Legis Stud. Author manuscript; Available in PMC 2024 Oct 29. (PMC7616756; doi:10.1080/13572334.2024.2411480)
Supplement: Supplemental material [file EMS199603-supplement-Supplemental_material.zip › Appendix_3.docx]

# **Appendix 3 – Results of the multiple-choice questions from the users’ questionnaires**

*Question 3 -* The context in which the evaluation of (x) took place.

*Question 4 -* In your opinion, has the evaluation of (x) had an impact, and if so, in what way?

*Question 5 -* The quality of the evaluation of (x)

*Question 6 -* The interaction between the researchers and the stakeholders in the evaluation of (x)

*Question 7 -* What factors do you think played a role in the impact generated by the evaluation of (x)?
