# Supplementary material for: Case studies on the impact of ex-post legislative evaluations in Dutch healthcare: a within and cross-case analysis
Source: J Legis Stud. Author manuscript; Available in PMC 2024 Oct 29. (PMC7616756; doi:10.1080/13572334.2024.2411480)
Supplement: Supplemental material [file EMS199603-supplement-Supplemental_material.zip › Appendix_4.docx]

# **Appendix 4 – Overview of the data results for each case**

# **Evaluation of the Youth Act (publication date: January 2018)**

| **Impact within legislation** | **Source** |
| --- | --- |
| The evaluation did not result in changes to the legal text. | None |
| **Impact within the political sector** | |
| Hearing/roundtable with municipalities, institutions, and clients initiated by the House of Representatives (2018) | Document analysis |
| Additional questions to the Minister from political parties (13 March, 2018)  Minister's response to additional questions (9 May, 2018) | Document analysis |
| Letter from the Standing Committee on the Evaluation of the Youth Act to the Minister (23 October, 2018)  Letter from the Minister regarding the evaluation of the Youth Act in response to the letter from the Standing Committee on 23 October, 2018 (28 November, 2018) | Document analysis |
| A debate was held in the House of Representatives regarding the evaluation of the Youth Act (21 June, 2018) | Document analysis |
| Additional questions from political parties PvdA and SP regarding the establishment of the Youth Authority (1 February, 2019)  Response from the Minister to the Senate (13 March, 2019) | Document analysis |
| Motion regarding recommendation two, mapping out waiting lists in youth care (27 June, 2023) | Document analysis |
| During a later political debate in the House of Representatives on the Reform Agenda for youth care, the PVV referred to the evaluation of the Youth Act in relation to the combination of decentralisation and budget cuts (27 June, 2023) | Document analysis |
| **Impact within policy circles** | |
| The Minister responded substantively (particularly policy-wise) to the evaluation in a letter to the Senate (30 January, 2018). It was indicated that the policy response to the interim evaluation of the Youth Act would follow in April to the Chamber in the form of a new programme entitled "Care for Youth." | Document analysis |
| The inspectors drafted a report based on this evaluation. This report was submitted to the House of Representatives by the Minister and Legal Protection (22 May, 2018). | Document analysis |
| The evaluation provided a justification for subsequent actions taken by the Minister, such as the launch of the "Care for Youth" Action Program on 16 April, 2018, which was developed in response to, amongst other things, the interim evaluation of the Youth Act. | Document analysis and focus group with researchers |
| "In the oversight domain, the evaluation internally assisted in familiarising colleagues from other domains who suddenly had to deal with youth-related issues with the problems surrounding the Youth Act. Externally, the evaluation contributed to making choices for a number of focal points, such as the forced framework and the child protection chain." | Focus group with users |
| "Certain bottlenecks identified in the evaluation are now being readdressed in the reform agenda." | Focus group with users |
| **Impact within the legal field** | |
| References to the evaluation in legal publications, such as:  ‘De Jeugdwet: tijd voor een evaluatie’ in Gst. 2017/120 M. Bruggeman (17-8-2017) ‘Drie ingrepen om de jeugdzorg te redden in [JusVer 2019/6.3 – I.E. Weijers – (1-12-2019](https://www.legalintelligence.com/documents/33408139?srcfrm=basic+search&docindex=30&stext=evaluatie%20jeugdwet)) ‘Zes jaar later: met z’n allen verstrikt geraakt in het stelsel?!’ in Tijdschrift voor Jeugdrecht, [E. Lam](https://www.bjutijdschriften.nl/zoek?search_category=&search_journal_code=&search_kind=author&search_text=Lam&search_year=) en [I.J.M. Schepens](https://www.bjutijdschriften.nl/zoek?search_category=&search_journal_code=&search_kind=author&search_text=Schepens&search_year=) (2021) | Document analysis and focus group with researchers |
| "The daily legal practice does not show that anything has been done with the recommendations." | Focus group with users |
| **Impact within society more broadly** | |
| There were many messages and reactions on websites from a wide variety of parties, including professional organisations, industry associations, and advocacy groups. | Document analysis |
| Letter from joint sector associations to the House of Representatives. Position Paper Specialised Youth Care Branches (BGZJ) 'Roundtable Discussion Evaluation of the Youth Act 23 April, 2018'. In this, the industry associations expressed their main criticism of the interim evaluation (that insufficient attention was paid to crucial policy-related areas and laws that are associated with the functioning of the Youth Act and that the government must ensure that the most important conditions are met by actively fulfilling its system responsibility). | Document analysis |
| The evaluation has been well read in the field, many conclusions are recognisable, but some field parties do not agree with the findings. | Document analysis and focus group with users |
| "The evaluation has set a number of things in motion, such as the connection with the general practitioner and the reduction of closed youth care." | Focus group with users |
| In response to the findings of the interim evaluation, it is indicated that efforts have already been made to improve access. | Document analysis and focus group with users |
| The field is critical of the choice of topics in the evaluation and finds certain subjects to be missing or underemphasised. Amongst other things, the voice of professionals are missed. To amplify this voice, 15 professional associations conducted their own survey, resulting in recommendations. | Focus group with users and focus group with researchers |
| ‘"The evaluation has brought field parties closer together in their frustrations about the system." | Focus group with researchers |
| The Dutch Youth Institute states that the evaluation provides an agenda for a joint approach by municipalities, care providers, and clients to further advance the transformation. | Document analysis |
| "The impact varies for each municipality. In some municipalities, the evaluation has had significantly more influence than in others. The impact is less clearly observable at the municipal level than at the policy or political level. Some administrators have addressed specific statements in the evaluation with researchers during meetings following the evaluation." | Focus group with researchers |
| **Context in which the evaluation took place** | |
| **Evaluation initiative and function**  The initiative for the evaluation was taken by the House of Representatives. In the autumn of 2013, the House of Representatives advanced the original evaluation period from 5 to 3 years by amendment. Subsequently, in the spring of 2016, they requested via a motion to designate this as an interim evaluation because it concerned the initial phase of a transformation process. The evaluation after five years could thus be considered in conjunction with the evaluation of other complementary laws. The evaluation could be used to strengthen the Minister's information position.  Subsequently, in the spring of 2016, the House of Representatives requested via a motion to designate the evaluation as an interim evaluation.  The assignment was as follows: 'The ultimate goal of this legislative evaluation is to provide insight into the effectiveness and (side) effects of the Youth Act in practice. Given both the scale and impact of the systemic change, this initial evaluation can only lead to preliminary conclusions. This evaluation can serve as the basis for monitoring the transformation periodically in the coming years and thus mapping out how municipalities and providers take on their new tasks over a longer period, and whether parents and children are better off in the new situation than before the systemic change. The evaluation research should generate more knowledge than is available based on previous studies.' | Document analysis  Focus group with users  Document analysis |
| **Political and societal influence**  Youth care is a broad and dynamic domain with many actors and a complex system that is politically and financially influenced. There has been a continuous struggle over the financial resources allocated to youth care.  At the time of the evaluation, a new government came into office, with a new minister who had their own agenda.  The evaluation took place concurrently with other research, causing it to be 'overshadowed.'  From the questionnaire sent to the field, it appears that there was discussion about the Youth Act, particularly in the healthcare sector, but also in policy and political circles.  The Social Domain Transition Committee urgently advises, in its fourth progress report, to consider and evaluate the Social Support Act, the Youth Act, and the Participation Act in conjunction due to their complementary nature. Furthermore, three years is deemed to be too short to form a definitive judgment on a law that brings about a transformation over several years. Consequently, there is a request for the government to consider the evaluation of the Youth Act after three years as an interim evaluation and to conduct the regular evaluation after five years in conjunction with that of the Social Support Act and the Participation Act. | Focus group with users and questionnaire with users  Focus group with users and questionnaire with users  Document analysis |
| **Openness to the evaluation results**  "The Minister was curious about the angles the evaluation could provide."  "The Minister wanted an interim assessment and control." | Focus group with researchers |
| **Quality factors that could have influenced the impact** | |
| **Composition and independence of the research group**  Five ZonMw referees rated the research group as 'good' and 'very good'. According to the referees, the research group is broadly composed, knowledgeable, and experienced.  Respondents from the field questioned whether the evaluation was independent and whether the Minister/political sector wanted a fair evaluation due to the limited design of the evaluation assignment. | Document analysis  Focus group with users |
| **Research design**  The evaluation consisted of a combined legal and empirical study. The design was partly based on two initial notes from a legal perspective and the Dutch Youth Institute. The evaluation focused on the implementation of the Youth Act, the direction of development, improvement possibilities, and the efficiency of the legal framework.  The researchers' approach was to think along with the law, maintain peace, and give the transformation process time.  "From the field, there was a need to evaluate the system as a whole; that's where they encountered the most issues in practice." | Document analysis  Focus group with researchers and  focus group with users |
| **Quality and content of the evaluation**  The project proposal was evaluated by five referees from ZonMw based on quality (objective and question-tasking, plan of approach, project group, and feasibility). The summary quality assessment was 'very good', 'good', and 'sufficient'.  The final report was discussed by the ZonMw Regulatory Evaluation Committee, and they gave a positive recommendation on the report. However, it was noted that the findings and/or recommendations could have been more assertive.  The report contained 21 recommendations that were not addressed to specific recipients.  The field considers it important to address recommendations, for example, to municipalities, so that the appropriate party feels addressed and takes responsibility, but can also be held accountable.  According to the field, the recommendations contained few concrete hooks, which made it difficult to implement in practice.  The early evaluation also meant that many points did not yet have solid findings or statements because the law had not yet crystallised in practice. This also led to a cautious formulation of certain results and/or recommendations.  According to field parties, certain topics were described less concretely and more reflectively, whilst other topics either lacked depth or were not covered. A common remark during the presentation of the results in the field by the researchers was that the evaluation did not address the budget cuts associated with the introduction of the Youth Act, which the field considered to be problematic.  Sector associations felt that the evaluation lacked connection to other laws such as the Education Act, Social Support Act, Participation Act, Long-term Care Act, and the Health Insurance Act. They also believed that there was no consideration of the connection between the Youth Act and the judicial chain, not to mention that there was no specific attention paid to young people with developmental issues or disabilities. The industry associations agreed with the conclusions but felt that the evaluation had too narrow a focus.  From the questionnaire sent to the field, most of the respondents rated the quality of the research group and the quality of the evaluation as being good and neutral, respectively.  According to the field, the evaluation contained a good summary. | Document analysis  Document analysis  Document analysis and  focus group with users  Focus group with users  Focus group with researchers  Focus group with users, document analysis, and questionnaire with researchers  Document analysis  Questionnaire with users  Focus group with users |
| **Interactional factors that could have influenced the impact** | |
| ***Interaction between researchers and the commissioner or participants***  During the research, there was ample opportunity for input from the field.  The report was discussed at regional and national 'round tables' with municipalities, institutions, and clients, after which a balanced policy response could be provided.  It was difficult to implement recommendations from the evaluation. According to some, there was not enough discussion. From clients’ perspective, there should have been more involvement with users and consumers.  From the questionnaire distributed to the field, it appears that people were both involved in the implementation and informed about the results, but they were either less or barely involved in the preparation, design, completion, and meetings.  According to one researcher, stakeholders were involved in discussions, different actors were separately surveyed through surveys and discussions, and attention was generated for the final report together with the clients. | Focus group with users  Document analysis  Focus group with users  Questionnaire with users  Questionnaire with researchers |
| ***Presentation and availability of the research results***  The evaluation report was published on various platforms including the websites of ZonMw, the Dutch Government, the Dutch Youth Institute, and Nivel. Furthermore, the legal experts from the evaluation research group wrote an article for the Journal for Family and Youth Law (FJR 2018/46) entitled "First evaluation of the Youth Act from a legal perspective".  According to the field, there was little publicity around the evaluation.  After the completion of the evaluation, researchers, at the initiative of the Ministry of Health, Welfare and Sport (VWS), travelled across the country to share the results, and a public congress was organised.  On 12 February, a national roundtable meeting was held in Utrecht. The researchers presented the results of the first evaluation of the Youth Act, followed by a brief explanation of the Action Programme for Child Abuse & Domestic Violence. After these two plenary presentations, around 100 representatives from municipalities, provider advocacy organisations, professionals, clients/experienced individuals, and the government engaged in discussions at eight thematic tables regarding the evaluation of the Youth Act and were able to contribute their input for both the Youth Care programme and Child Abuse and Domestic Violence programme.  Following the evaluation, the researchers held discussions with various stakeholders across the Netherlands, including networks of aldermen and youth care organisations, to discuss the findings of the evaluation. The researchers reported that the responses were varied, ranging from positive to critical comments concerning the choice of topics.  Not all of the researchers saw impact as being the responsibility of the researchers. After completing the research, the researchers let it go. | Document analysis  Focus group with users  Questionnaire with researchers  Document analysis  Document analysis  Focus group with researchers |
| ***Timing***  In the autumn of 2013, the House of Representatives adjusted the original evaluation period from five to three years by amendment due to the complexity of the law, which involved a large package of tasks in the field of care and assistance to a vulnerable group of young people being transferred to municipalities.  At this preliminary stage, the law had yet to fully crystallise, and thus no solid findings or statements could be made. The signals regarding bottlenecks or systemic problems could also be easily dismissed by the early evaluation because only two years had passed. | Document analysis  Focus group with users and focus group with researchers |

# **Evaluation of Wkkgz (publication date: January 2021)**

| **Impact within legislation** | **Source** |
| --- | --- |
| The Minister intended to amend and/or expand the Wkkgz and the Implementation Decree in response to various recommendations from the legislative evaluation. This was coordinated on certain points with, amongst others, the Healthcare Inspectorate (IGJ), the Dutch Healthcare Authority (NZa), and field parties. | Document analysis and focus groups |
| **Impact within political circles** | |
| The Minister referred in a response to parliamentary questions on another topic (sexual misconduct in youth care) to the evaluation of the Wkkgz. | Document analysis |
| Various political parties asked additional questions to the Minister prior to the government's response. The Standing Committee on the VWS asked the Minister questions about the response to the evaluation of the Wkkgz. | Document analysis |
| **Impact within policy circles** | |
| The Minister responded substantively to the evaluation in a letter to the House of Representatives (1 July 2022). | Document analysis |
| The Minister acted on various recommendations, including conducting further research, providing additional support to the field, adjusting policy regulations, and holding further discussions with other parties, including the IGJ. Some recommendations were not adopted because the Minister did not consider the proposed situation desirable, preferred to maintain the current state of affairs, or believed that the recommendation was directed at other parties. | Document analysis |
| The inspectorate was eager to engage in discussions with field parties to explore how it can ensure that such matters are brought to the attention of care providers (even) more effectively. This also applied to stimulating the dissemination of lessons learned amongst care providers themselves. | Document analysis |
| Along with the IGJ, it was explored whether the lack of clarity regarding subcontracting, as outlined by the researchers but for which no recommendation was made, posed a problem for supervision practice. If so, both the extent of this problem and whether the IGJ could manage with the current regulations in the Wkkgz were examined. | Document analysis |
| Within policy departments of the VWS, the evaluation was consulted weekly as a reference. The evaluation provided guidance, for example, in understanding the perspective of healthcare providers. | Focus group with users |
| **Impact within the legal domain** | |
| Law firm KBS Advocaten posted a message about the results of the Wkkgz on their website. The University of Amsterdam also posted a message about the results of the Wkkgz on their website, with specific reference to the two legal researchers who worked there. | Document analysis |
| The outcomes of the evaluation regarding complaints and dispute resolution were the subject of discussion during an accredited course for legal professionals entitled "Dispute Resolution under the Wkkgz: Theory in Practice". | Document analysis |
| **Impact within society more broadly** | |
| Several industry associations responded in a letter to the House of Representatives regarding the evaluation. In one of the letters, it was highlighted that certain topics were not investigated in the evaluation. | Document analysis |
| Various organisations posted articles about the results of the evaluation, including industry associations, a management consultancy firm, a knowledge and learning network, and a healthcare news website. | Document analysis |
| "Dispute resolution bodies have approached each other following the legislative evaluation to engage in more collaborative discussions." | Focus group with users |
| Research institute Nivel conducted in-depth research on involving patients and their relatives after an incident within a Dutch hospital, at the request of the IGJ, partly in response to the evaluation. | Document analysis |
| **Context in which the evaluation took place** | |
| **Evaluation initiative and function**  The Wkkgz has been fully in effect since 1 January, 2017. It regulates healthcare quality and complaint procedures, partially replacing existing legislation that was evaluated in the past. In addition to familiar provisions, the law also introduced new obligations. The Wkkgz is a broad and organisational law that is applicable to all healthcare providers.  To effectively monitor the law, the Minister commissioned a baseline measurement of the Wkkgz in 2016. Subsequently, an annual monitor of the Wkkgz was conducted, focusing on developments in complaints and disputes.  Five years after its enactment, the Wkkgz was evaluated based on the evaluation clause in the law.  In the assignment of the evaluation, it was stated that the evaluation of the Wkkgz aimed to report on both the effectiveness and effects of the law in practice. Researchers were tasked with answering this central question comprehensively, whilst also taking into account several sub-questions formulated into four categories: the scope of the law, accessibility of complaint procedures, monitoring and promoting healthcare quality, and supervision by the Healthcare Inspectorate. | Document analysis  Document analysis  Document analysis  Document analysis |
| **Political and societal influence**  In the form of a commitment to the Senate, the Minister announced that the evaluation should examine the extent to which the goals of the law were being achieved. The evaluation would compare the situation during the baseline measurement and the conducted monitors, and specific questions from the Senate would be addressed.  Later, the Minister added several additional commitments regarding the right to lodge complaints, the obligation to provide information in the event of incidents, the supervisory role of the IGJ, and Article 20 of the Wkkgz.  The evaluation took place during the COVID-19 pandemic.  The responses to user surveys indicated that there had been discussion about the Wkkgz, particularly in policymaking circles but also within the fields of healthcare and politics. | Document analysis  Document analysis  Focus group with users and questionnaire with users  Questionnaire with users |
| **Openness to the evaluation results**  / |  |
| **Quality factors that influenced the impact** | |
| **Samenstelling en onafhankelijkheid van de onderzoeksgroep**  The project team received ratings of "excellent" and "good" in the ZonMw referee comments. | Document analysis |
| **Research Design**  The evaluation consisted of a combined legal and empirical research approach. The researchers chose to organise an expert meeting at the beginning of the evaluation to identify the key issues and conducted the evaluation with a particular focus on five specific healthcare sectors.  This broad approach, according to the stakeholders, whilst affording a general impression of the functioning of the law also prevented in-depth analysis. As a result, the policy domain lacked sufficient guidance to formulate responses on certain topics.  Several stakeholders argued that the report primarily targeted policymakers. Consequently, it may be less suitable for clients and professionals, even though they also need to engage with it. The report was perceived as overly abstract by the field, which may have meant that stakeholders did not fully understand the implications of the legislative evaluation. Professional associations could assist in this regard.  The researchers indicated that this report was indeed intended for policymakers rather than the field. | Document analysis  Focus group with users  Focus group with users  Focus group with researchers |
| **Quality and Content of the Research Report**  The evaluation yielded 32 recommendations, directed at seven different groups of addressees: legislators (16), the ministry (15), the regulatory authority (8), professional associations (8), patient organisations (4), healthcare providers (2), and the field (1).  Stakeholders reported that they would act upon the evaluation results when the recommendations were concrete and feasible.  During a final advisory committee meeting in which the draft version of the research report was discussed, it was noted that the report was thorough, but that there were some issues regarding consistency in the structure, the distinction between main and secondary issues, and in terms of the alignment between the findings and recommendations.  The final report was reviewed by the Evaluation of Legislation Committee of ZonMw, who provided a positive assessment of the report. However, it was observed that the research had a low response rate, was focused more on professionals than patients, and lacked sharpness in its recommendations on topics that concerned practice, due to the ways in which the questions were formulated.  According to the feedback from the field via a questionnaire, most respondents rated the quality of the research group and the evaluation as being good and neutral. | Document analysis  Focus group with users  Document analysis  Document analysis  Questionnaire with users |
| **Interactional factors that could have influenced the impact** | |
| **Interaction between researchers and the principal or participants**  In the draft version of the report, it was not specified who should be primarily responsible for the recommendations. The researchers adjusted this in the final version based on feedback from the advisory committee.  The Minister stated that field parties would be involved in the actions resulting from the policy response to the evaluation of the Wkkgz.  Regardless of how the evaluation was received (through participation, a webinar, or other communication), the field emphasised that it initiated processes. This was recognised by the field because, for example, dispute resolution bodies approached each other to collaborate more as a result of the evaluation.  Because the evaluation took place during the COVID-19 pandemic, the researchers interacted with respondents in a different (digital) manner. According to one researcher, this impacted upon the research process, especially in terms of how they interacted with respondents during the study. For example, physical meetings were not possible for a long period, so they took place digitally, which was a new experience. The circumstances related to the COVID-19 pandemic also delayed the policy response from the VWS to the evaluation.  According to the (legal) field, involvement in the research led to a greater interest in the results and the follow-up of the research, and there was a feeling that participation in the evaluation research could to some extent influence the outcome(s). Field parties mentioned that being involved also provided them with food for thought, even during the evaluation. The evaluation itself was already called 'an intervention in itself'. The exchanges that arose during the focus group discussions made the involved parties think and were incorporated into their own organisation. These insights created awareness and sometimes even helped to initiate processes.  According to the researchers, at the start of the project, various parties discussed which (sub)topics would be examined in the context of the evaluation, or where the focus would lie. The field was involved in the evaluation from various health sectors, resulting in a lot of interaction during the research, and there was a lot of contact with policymakers. The addressees were explicitly mentioned in the recommendations.  According to the field's responses in the questionnaire, they were involved in the preparation and execution, and were informed about the outcomes and meetings, but were either less or barely involved in the design and conclusion. | Document analysis  Document analysis  Focus group with users  Focus group with users  Focus group with researchers and questionnaire with researchers  Questionnaire with users |
| ***Presentation and availability of the research results***  The evaluation report was published on the websites of ZonMw, the Dutch government, Nivel, and some industry organisations.  The researchers distributed the evaluation report to stakeholders.  The researchers organised a webinar to discuss the results of the evaluation with field parties and professionals.  Four researchers involved in the evaluation authored an article for the Journal of Health Law entitled (translated: “Three Core Themes from the Evaluation of the Wkkgz)" (2021).  A network of patient councils in healthcare requested an accessible version of the Wkkgz evaluation report. According to them, the current document contained a lot of important information for patients and patient councils. "The current document is very extensive and contains a lot of legally technical information. This makes it mainly suitable for policymakers, whilst people in practice need to work with it. We request the committee members to ask the Minister for a more accessible version of the research report. It should be suitable for patient councils and staff representation to have broad discussions about good care within the healthcare organisation.” | Document analysis  Questionnaire with researchers  Document analysis and questionnaire with researchers  Document analysis  Document analysis |
| ***Timing***  ***/*** |  |

# **Evaluation of the Embryo Act (publication date: February 2021)**

| **Impact within legislation** | **Bron** |
| --- | --- |
| The Minister wrote in his response that the Embryo Act would be adjusted in two aspects based on the evaluation. | Document analysis, focus group with users and focus group with researchers |
| Two governing parties were drafting a private members' bill that aligned with a recommendation from the legislative evaluation. | Document analysis and focus group with researchers |
| From the two main human-animal combinations currently not regulated, the Minister, in response to the evaluation, intended to regulate one (cybrid) via the Embryo Act and exclude the other (hiPSC-chimera) from the Embryo Act. This aligned with the recommendation from the legislative evaluation. | Document analysis |
| **Impact within political circles** | |
| Various parties posed additional questions to the Minister. | Document analysis |
| In a coalition agreement in preparation for a new term of office, it was described what would be done with specific recommendations from the legislative evaluation. | Document analysis |
| The House of Representatives held an online consultation on the 'Amendment Law abolishing the prohibition on the creation of embryos. | Document analysis |
| “The government has left some topics untouched and not adopted recommendations, such as germline modification." | Focus group with researchers |
| **Impact within policy circles** | |
| The Minister responded substantively to the evaluation in a letter to the House of Representatives. | Document analysis |
| The cabinet, in accordance with the interim evaluation, requested advice from the Health Council regarding both the desirability and acceptability of extending the fourteen-day limit to twenty-eight days (recommendation seven) and how to deal with a developmental limit for ELS (recommendation eight). This advice was provided on 31 October, 2023. | Document analysis |
| In his response to the evaluation, the Minister stated that the Netherlands would participate in the dialogue on germline modification at the European level. If there was a discussion about the possibilities of preclinical research, then the Minister stated that they would strive to prevent European regulations or treaties from complicating preclinical research, which is in line with the recommendation of the legislative evaluation. | Document analysis |
| The ministry was taking up the recommendations and was looking into how they could be followed up on. | Document analysis |
| **Impact within the legal field** | |
| The evaluation was included in the course material for the course 'Legal Issues Surrounding the Beginning and End of Life' (3864REC7KY) at the University of Amsterdam. | Document analysis |
| **Impact within society more broadly** | |
| The Lindenboom Institute wrote the report entitled 'Annotations on the third evaluation of the Embryo Act,' in which criticism is offered on the recommendation of the evaluators to abolish the prohibition on culturing embryos. | Document analysis |
| Stakeholders posted the evaluation on their website, whilst references to the evaluation were made in articles published in the Healthcare Journal, namely: 'Embryo Act Amended for the First Time Since 2002' or 'Is Germline Modification Ethically Responsible?' by G. de Wert and W. Dondorp, published in the NtvG on November 28, 2022. | Document analysis |
| **Context in which the evaluation took place** | |
| **Evaluation initiative and function**  The Embryo Act was evaluated twice before (in 2006 and 2012, respectively). This was thus the third evaluation of the Embryo Act.  In the ministry's assignment letter, it was stated that the third evaluation should broadly address the general functioning of the Embryo Act. Additionally, the research would be used to focus on themes identified as bottlenecks in previous legislative evaluations (which had not yet been resolved) and/or that required further attention due to medical-scientific or societal developments.  The CER chose not to include the societal perspective in the research. | Document analysis, focus group with users and focus group with researchers  Document analysis  Document analysis |
| **Political and societal influence**  The domain covered by the Embryo Act was delimited, and stakeholders and policy officials were familiar with each other.  By conducting evaluations periodically, alignment with everyday practice could be continually sought. Discussions were also held following the evaluation.  New developments in embryo research increased the relevance of topics mentioned in previous evaluations and required adjustments to the law. These topics were important for the selection of subjects for the legislative evaluation.  During the evaluation, changes in staff working on this dossier occurred at the VWS. It was noted from the field that they felt they had to start over completely with each of these changes.  Whether recommendations were followed up on and implemented largely depended on political positions, the composition of the coalition, and the agreements of the cabinet.  In the political arena, certain subjects, such as the specific cultivation of embryos, were simply not up for discussion. This meant that, for example, policy could not proceed with the outcomes of the societal dialogue.  The political context did not influence how the evaluation assignment was established because there was simply a need for scientific and independent advice. At most, emphasis could be placed on certain aspects.  The questionnaire results indicated that discussions about the Embryo Act primarily took place in policy and political circles, with less emphasis within the healthcare sector. | Focus group with users and focus group with researchers  Focus group with users, focus group with researchers and questionnaire with users  Focus group with researchers  Focus group with users  Focus group with users  Focus group with researchers  Focus group with researchers  Questionnaire with users |
| **Openness to evaluation results**  / |  |
| **Quality factors that could have influenced the impact** | |
| **De composition and independence of the research group**  The project group was rated 'excellent' in the referee comments and they were considered experts in the field.  Only a small group of people were sufficiently qualified and well-versed in the field to be able to conduct such evaluations (field focus group). Therefore, the field assumed that the evaluation was conducted scientifically and was well substantiated.  The authority of the research group was important for the impact.  Researchers had to safeguard their independent positions as experts and avoid becoming part of the political discussion. To ensure this independence, it was important to strike the delicate balance between various interests, moral positions, and societal perspectives. A critical debate within a multidisciplinary research group contributed towards balanced recommendations. | Document analysis and focus group with users  Focus group with researchers  Focus group with researchers |
| **Research design**  The evaluation consisted of a combined legal and ethical investigation. The researchers chose to focus on six themes because most of the related problems and bottlenecks identified in previous evaluation studies had yet to be resolved, and questions about the future viability of the law also particularly related to those themes.  The project proposal was evaluated by three referees with respect to its quality (objective and question-tasking, approach plan, project group, and feasibility). The summary quality assessment was rated 'excellent' by one referee, 'good' by one referee, and 'moderate' by one referee.  To achieve maximum impact, researchers during an evaluation should look at bottlenecks and gaps in practice and be aware of debates or pressing issues in society.  There was a discussion within the Committee for Evaluation of Regulation (CER) of ZonMw about whether societal perspectives should form part of the evaluation. Gaining information about these perspectives was very important for the ministry. Ultimately, the CER chose not to include this perspective in the research. It was proposed to look more at societal legitimisation in the next assignment, in order to supplement what had already been done. | Document analysis  Document analysis  Focus group with researchers  Document analysis  Document analysis |
| **Quality and content of the research report**  Fifteen recommendations were made, with 12 addressed to the legislator, five to the VWS, one to the research field, and one to KLEM.  The final report was discussed in the CER of ZonMw, who provided a positive recommendation about the report. However, it was advised to adjust certain formulations in the recommendations.  The questionnaire results indicated that both the quality of the evaluation and the research group were rated very good to good. | Document analysis  Document analysis  Questionnaire with users |
| **Interactional factors that could have influenced the impact** | |
| **Interaction between researchers and commissioners or participants**  During the evaluation, the researchers engaged with various individuals who held different viewpoints. The selection of respondents was not based on their specific stances but rather on their expertise and experience.  The field experts were closely involved in discussions pertaining to legislation and regulations, such as private members’ bills, as these developments had significant potential for societal impact. These initiatives were, in part, derived from legislative evaluations. Involving experts during the evaluation process lent weight to crucial points for stakeholders in the field. Understanding current issues was essential for an effective evaluation.  In addition to involving field experts, there was also emphasis at that time within the policy domain on societal dialogue to better understand public perspectives on certain Embryo Act-related topics, such as the specific cultivation of embryos. This provided insight into these necessary debates. However, not every topic was conducive to this approach, and careful consideration of terminology was crucial. Attention to societal consensus likely also played a key role in the evaluation assignment with respect to outlining prerequisites.  According to respondents from the field, there had been a lot of discussion in recent years without corresponding action, although this was also viewed as a valuable learning experience. Researchers directed their recommendations towards the appropriate recipients.  The results from the user questionnaires indicated that they were involved in the preparation, design, and execution as well as being informed about the results, but were either less or barely involved in the finalisation and meetings.  According to the responses from the researchers, in the case of legislative evaluations, a cabinet response to recommendations was necessary, given parliamentary involvement. This subject was already garnering societal and political attention, and thus necessitated fewer additional actions. | Focus group with researchers  Focus group with users  Focus group with users  Focus group with users  Questionnaire with users  Questionnaire with researchers |
| ***Presentation and availability of the research results***  The evaluation report was published on the websites of various organisations, including ZonMw, the Dutch government, the Central Committee on Research Involving Human Subjects (CCMO), and Maastricht University.  An article in TvGr entitled ‘Kiembaanmodificatie: goed geregeld in de Embryowet?’ M. Spaander, M.C. Ploem, G.M.W.R. de Wert (2023).  According to the researchers, publications were written, and a conference was organised at the end of the trajectory. | Document analysis  Document analysis  Questionnaire with researchers |
| ***Timing***  The evaluation took place in 2020. Originally, the evaluation was scheduled for 2017, but it was postponed for several years by the Minister due to ongoing legislative changes at that time (which stemmed from the two previous legislative evaluations). | Document analysis |
| ***Other***  Researchers took the implementation opportunities into account when formulating their recommendations, for example by ensuring sufficient policy discretion in the recommendations directed at the government (such as the recommendation to seek advice from the Health Council).  Carefully formulated recommendations directed at the appropriate recipient are important. Recommendations must be relevant and substantively strong and can lead to changes in policy, legislation, scientific practices, and healthcare. The researchers were acutely aware that the manner in which the recommendations were formulated was crucial. For example, it was important to retain sufficient policy discretion in recommendations directed at the government. | Focus group with researchers  Focus group with researchers |
